# Triple Gram-negative bacterial endophthalmitis following intravitreal injection

**DOI:** 10.1186/s12348-023-00376-9

**Published:** 2024-02-09

**Authors:** Samy Zaher, Hector Rodriguez-Villalobos

**Affiliations:** 1 Department of Ophthalmology, Cliniques Universitaires Saint-Luc, Brussels, Belgium; 2 Department of Microbiology, Cliniques Universitaires Saint-Luc, Brussels, Belgium

**Keywords:** Intravitreal injection, Aflibercept, Endophthalmitis, *Pseudomonas stutzeri*, *Stenotrophomonas maltophilia*, *Ochrobactrum anthropic*, Multi-drug resistance

## Abstract

**Purpose:**

To describe a puzzling case of endophthalmitis caused by three unusual bacteria after intravitreal injection, its outcome, and underlying questions.

**Findings:**

A 70-year-old female patient was diagnosed with acute endophthalmitis following intravitreal aflibercept injection for age-related macular degeneration. A standard tap and inject procedure was performed. Microbiological analyses on the anterior chamber and vitreous samples yielded the presence of three non-fermenting Gram-negative rods: *Pseudomonas stutzeri*, *Stenotrophomonas maltophilia*, and *Ochrobactrum anthropi*. The outcome was favorable after intravitreal injections of vancomycin and ceftazidime, with an almost complete recovery of the visual acuity to its baseline level. No potential source of infection was identified.

**Conclusion:**

Endophthalmitis following intravitreal injection can be caused by a wide variety of bacteria, including some rare Gram-negative species. They can sometimes co-exist in a single patient, but their virulence may vary greatly. Due to the variable antibiotic susceptibility and frequent multiresistance associated with non-fermenting Gram-negative rods, a prompt microbiological approach is required. Favorable outcome can be achieved with standard management.

## Introduction

Intravitreal injections of anti-vascular endothelial growth factor (anti-VEGF) have become a cornerstone treatment for various ophthalmic diseases, particularly wet age-related macular degeneration (AMD). Endophthalmitis is a rare and serious complication following the procedure, with a reported incidence of approximately 1 in 2,000 injections [1]. It is most commonly associated with Gram-positive microorganisms, such as *Staphylococcus* species [2]. Gram-negative bacteria are often considered to be more virulent. We here report a case of acute polymicrobial endophthalmitis, caused by an association of three uncommon non-fermenting Gram-negative bacteria, following an intravitreal injection of aflibercept for AMD.

## Case presentation

A 70-year-old woman with a history of regular anti-VEGF intravitreal injections in both eyes for wet AMD was referred to our ophthalmic emergency department by her treating ophthalmologist for decreased visual acuity, ocular pain, and photophobia in her right eye for one day. Six days earlier, she underwent bilateral aflibercept intravitreal injections without any complications. She had a history of pars plana vitrectomy and cataract surgery for an epiretinal membrane in the fellow left eye fifteen years earlier. Her medical history was only relevant for treated hypercholesterolemia. Best-corrected visual acuity was counting fingers in the right eye and 20/25 in the left eye. Intraocular pressure was 14.5 mmHg and 20 mmHg in the right and left eyes respectively. Mild diffuse conjunctival injection, 3 + anterior chamber cells and a 3 + nuclear cataract, with no fibrin or hypopyon, were observed in the right eye on anterior segment examination. Posterior segment examination showed 3 + vitreous cells and fundus examination, although hazy, appeared unremarkable with no sign of retinitis. B-scan ultrasonography revealed a heterogeneous vitreous cavity with a well-attached retina. A diagnosis of acute post-injection infectious endophthalmitis was made. The patient underwent anterior chamber and vitreous taps under retrobulbar anesthesia, followed by intravitreal injections of vancomycin (1.0 mg/0.1 mL) and ceftazidime (2.25 mg/0.1 mL), along with a subconjunctival injection of betamethasone. Topical dexamethasone 0.1% hourly was initiated. The microscopic examination of anterior chamber and vitreous samples by Gram stain did not show any microorganism. Aerobic cultures of both samples, processed separately, returned positive after three days of incubation for three non-fermenting Gram-negative rods identified by MALDI-TOF mass spectrometry as *Pseudomonas stutzeri*, *Stenotrophomonas maltophilia* and *Ochrobactrum anthropi*. Both *S. maltophilia* and *O. anthropi* exhibited high levels of antibiotic resistance, including ceftazidime (Table 1). Forty-eight hours after presentation, significant clinical improvement was observed, with an increase in visual acuity to 20/200 and reduction in anterior chamber inflammation to 1 + cells. After three days, topical treatment was gradually tapered, and at the 1-week follow-up visit, the patient’s pain had resolved and her best-corrected visual acuity had improved to 20/100, slightly lower than the pre-injection baseline of 20/60 for this eye. Slit lamp examination showed no conjunctival injection, residual 0.5 + anterior chamber cells and 1 + vitreous cells. The fundus was unremarkable, except for a known area of macular geographic atrophy and a small peripheral inferotemporal subretinal hemorrhage. After two weeks, the patient was referred back to her treating ophthalmologist for continued follow-up of intraocular inflammation and resumption of anti-VEGF treatment for intraretinal fluid recurrence. Future cataract surgery will be discussed with the patient.

**Table 1:** Antibiotic susceptibilities of the three bacteria

	*Pseudomonas stutzeri*	*Stenotrophomonas maltophilia*	*Ochrobactrum anthropi*
Amikacin	S	**R**	S
Aztreonam	S	**R**	**R**
Ceftazidime	S	**R**	**R**
Ciprofloxacin	S	S	S
Colistin	S		S
Cefepime	S	**R**	S
Gentamicin		**R**	
Imipenem	S	**R**	S
Minocycline		S	S
Meropenem	S	**R**	S
Piperacillin	S	**R**	**R**
Piperacillin/Tazobactam	S	**R**	**R**
Ticarcillin	S	**R**	**R**
Ticarcillin/Clavulanate	S	S	**R**
Tobramycin	**R**	**R**	**R**
Trimethoprim/sulfamethoxazole		S	S

*S* Susceptible, *R* Resistant

## Discussion

The present case reports a rare instance of endophthalmitis following intravitreal injection caused by three uncommon Gram-negative bacteria, namely *Pseudomonas stutzeri*, *Stenotrophomonas maltophilia*, and *Ochrobactrum anthropi*. Gram-negative organisms are known to be of exogenous origin, as they are not normal commensal bacteria of the conjunctiva or periocular skin [3]. All three pathogens have been reported to be present in soil, contaminating surfaces and water sources, inside and outside of the hospital setting [4–6].

Several cases have been published of endophthalmitis caused by each of these organisms, mostly following cataract surgery. *S. maltophilia* is the most commonly reported pathogen, while *P. stutzeri* is the least common. *S. maltophilia* has also been reported in cases of endophthalmitis following trauma, pars plana vitrectomy and keratoplasty as well as endogenous endophthalmitis [7, 8]. *O. anthropi* is commonly associated with infections related to medical devices, such as indwelling catheters, or following recent vascular procedures, potentially causing endogenous endophthalmitis [5]. It has also been reported following pars plana vitrectomy and keratoprosthesis implantation [9, 10]. Only *S. maltophilia* has been reported in infections following intravitreal injections. Two outbreaks occurred in the context of shared freshly opened vials of bevacizumab, and one case followed aflibercept injection that was successfully treated with a tap and inject procedure [6, 11, 12].

These three bacteria can cause both acute and chronic forms of endophthalmitis. *S. maltophilia* more frequently presents in an acute manner, while *O. anthropi* is often associated with a low-grade chronic inflammation. In our case, the patient presented with acute endophthalmitis, characterized by moderate inflammation. Despite the co-infection with three different germs, the limited amount of intraocular inflammation could be attributed to probably low inoculum and the known limited virulence of the three bacteria [12–14]. Nevertheless, the infection presented acutely after 6 days and not as a delayed chronic inflammation.

When following cataract surgery, infections by each of these bacteria have usually been attributed to contaminated tubing or irrigating solutions [3, 15, 16]. In Boeke’s case report of *S. maltophilia* endophthalmitis following aflibercept injection, reusable irrigating solution was suspected to be the source of the infection [12]. Given the use of single-use products during the intravitreal injection procedure, including oxybuprocaine, isobetadine, aflibercept and the rinsing solution, a simultaneous infection with these three bacteria is difficult to explain and trace. The microbiological results were consistent and confirmed in both the anterior chamber tap and vitreous tap. Laboratory contamination of two samples, processed separately, by these three distinct germs appears highly unlikely. No source of infection could be identified at the treating ophthalmologist’s practice. Notably, there was no infection observed in the contralateral eye that underwent treatment at the same time or in any other patients treated on the same day. One hypothesis would be a breach in sterility during the procedure itself.

*S. maltophilia* and *O. anthropi* are considered as multi-drug resistant (MDR) organisms due to their broad spectrum of antibiotic resistance, but the reported resistance rates vary widely in the literature and seem to be evolving over time [17, 18]. Notably, Chen et al. described high resistance of *S. maltophilia* to ceftazidime and amikacin, which were historically considered as good therapeutic options [19, 20]. Ciprofloxacin, which has recently been highlighted as a good first-line antibiotic against *S. maltophilia* and is also a treatment of choice for *O. anthropi*, could have been used in our case [17, 21]. Additionally, as regularly described in the literature, trimethoprim/sulfamethoxazole would have been a good treatment option for both *S. maltophilia* and *O. anthropi*, despite recent mentions of high resistance to this antibiotic [17, 22]. While a wide range of antibiotic resistance has been reported for *P. stutzeri*, it has not been substantial in most published cases of endophthalmitis, including ours [3, 13].

Considering the moderate severity of the infection, our patient was treated with a non-surgical tap and inject approach rather than a primary pars plana vitrectomy. The treatment ultimately proved effective in resolving the infection within a few days. Resistance to ceftazidime or the use of vancomycin, a Gram-positive specific agent, without any topical or oral antibiotic, did not hinder a good clinical outcome of the infection. This has been described in other cases and can be explained by the fact that the intraocular concentration of antibiotics is often much higher than the minimum inhibitory concentration defining in vitro bacterial resistance. However, initial antibiotic resistance could potentially explain some instances of delayed recurrences [23]. Recurrences days or months after resolution of intraocular inflammation have often been reported with these particular bacteria, *mostly S. maltophilia*, and it cannot be formally ruled out at the moment. Long-term follow-up of these patients is thus mandatory. Nevertheless, most of them are associated with the implantation of an intraocular lens during cataract surgery, which usually requires removal [24].

While endophthalmitis associated with Gram-negative bacteria usually has a poorer outcome [23], infections caused by these specific organisms do not seem to have a less favorable prognosis than those caused by more typical Gram-positive bacteria [3, 18, 23].

## Conclusion

This case presents a rare instance of polymicrobial endophthalmitis caused by three uncommon non-fermenting Gram-negative bacteria, rarely associated with intravitreal injection. The source of the infection in such cases is probably related to the environment, soil and water but often remains unknown. A rapid microbiological approach is necessary due to the variable antibiotic susceptibility and emergent multidrug resistance among these bacteria. Nonetheless, the management and final outcome does not necessarily differ from that of infection caused by more usual bacteria.

## Data Availability

Not applicable.

## References

[1] Storey PP, Patel D, Garg S (2020). Endophthalmitis following intravitreal injection of anti-vascular endothelial growth factor agents. Can J Ophthalmol.

[2] Sachdeva MM, Moshiri A, Leder HA, Scott AW (2016). Endophthalmitis following intravitreal injection of anti-VEGF agents: long-term outcomes and the identification of unusual micro-organisms. J Ophthalmic Inflamm Infect.

[3] Handa S, Singh SR, Sharma B, Rana V, Bazgain K, Tekchandani U (2022). Cluster outbreak of pseudomonas stutzeri acute endophthalmitis following phacoemulsification: a report of 14 cases from North India. Indian J Ophthalmol.

[4] Alshahrani ST, Arevalo JF (2020). Chronic endophthalmitis caused by Pseudomonas stutzeri. Case Rep Ophthalmol.

[5] Berman AJ, Del Priore LV, Fischer CK (1997). Endogenous ochrobactrum anthropi endophthalmitis. Am J Ophthalmol.

[6] Agarwal AK, Aggarwal K, Samanta R, Angrup A, Biswal M, Ray P (2019). Cluster endophthalmitis due to Stenotrophomonas maltophilia following intravitreal bevacizumab: outcomes of patients from North India. Br J Ophthalmol.

[7] Chen KJ, Wang NK, Sun MH, Chen TL, Lai CC, Wu WC (2010). Endophthalmitis caused by Stenotrophomonas maltophilia. Ophthalmic Surg Lasers Imaging.

[8] Chhablani J, Sudhalkar A, Jindal A, Das T, Motukupally SR, Sharma S (2014). Stenotrophomonas maltophilia endogenous endophthalmitis: clinical presentation, antibiotic susceptibility, and outcomes. Clin Ophthalmol.

[9] Inoue K, Numaga J, Nagata Y, Sakurai M, Aso N, Fujino Y (1999). Ochrobactrum anthropi endophthalmitis after vitreous Surgery. Br J Ophthalmol.

[10] Chan CC, Holland EJ (2012). Infectious endophthalmitis after Boston type 1 keratoprosthesis implantation. Cornea.

[11] Beri S, Shandil A, Garg R (2017). Stenotrophomonas maltophilia: an emerging entity for cluster endophthalmitis. Indian J Ophthalmol.

[12] Boeke PS, Gottlieb JL (2019). Stenotrophomonas maltophilia endophthalmitis 1 month after intravitreal aflibercept. Retin Cases Brief Rep.

[13] Lalucat J, Bennasar A, Bosch R, Garcia-Valdes E, Palleroni NJ (2006). Biology of pseudomonas stutzeri. Microbiol Mol Biol Rev.

[14] Braun M, Jonas JB, Schonherr U, Naumann GO (1996). Ochrobactrum anthropi endophthalmitis after uncomplicated cataract surgery. Am J Ophthalmol.

[15] Akcakaya AA, Sargin F, Erbil HH, Yazici S, Yaylali SA, Mesci C (2011). A cluster of acute-onset postoperative endophthalmitis over a 1-month period: investigation of an outbreak caused by uncommon species. Br J Ophthalmol.

[16] Mattos FB, Saraiva FP, Angotti-Neto H, Passos AF (2013). Outbreak of Ochrobactrum anthropi endophthalmitis following cataract Surgery. J Hosp Infect.

[17] Ho MC, Hsiao CH, Sun MH, Hwang YS, Lai CC, Wu WC (2021). Antimicrobial susceptibility, minimum inhibitory concentrations, and clinical profiles of Stenotrophomonas maltophilia endophthalmitis. Microorganisms.

[18] Kannan NB, Kohli P, Shekhar M, Sen S, Lalitha P, Pai A (2022). Ochrobactrum anthropi: a rare cause of culture-proven acute post-operative cluster endophthalmitis. Ocul Immunol Inflamm.

[19] Chen KJ, Sun MH, Hou CH, Chen HC, Chen YP, Wang NK (2021). Susceptibility of bacterial endophthalmitis isolates to Vancomycin, ceftazidime, and amikacin. Sci Rep.

[20] Chang JS, Flynn HW, Miller D, Smiddy WE (2013). Stenotrophomonas maltophilia endophthalmitis following cataract surgery: clinical and microbiological results. Clin Ophthalmol.

[21] Kanjee R, Koreishi AF, Tanna AP, Goldstein DA (2016). Chronic postoperative endophthalmitis after cataract Surgery secondary to Vancomycin-resistant Ochrobactrum anthropi: case report and literature review. J Ophthalmic Inflamm Infect.

[22] Al-Jasser AM (2006). Stenotrophomonas maltophilia resistant to trimethoprim-sulfamethoxazole: an increasing problem. Ann Clin Microbiol Antimicrob.

[23] Chaudhry NA, Flynn HW, Smiddy WE, Miller D (2000). Xanthomonas maltophilia endophthalmitis after cataract Surgery. Arch Ophthalmol.

[24] Chiang CC, Tsai YY, Lin JM, Chen WL (2009). Chronic endophthalmitis after cataract Surgery secondary to ochrobactrum anthropi. Eye (Lond).

